# Single Cell–ICP–ToF-MS for the Multiplexed Determination of Proteins: Evaluation of the Cellular Stress Response

**DOI:** 10.1021/acs.analchem.3c02558

**Published:** 2023-08-11

**Authors:** Paula Menero-Valdés, Michail I. Chronakis, Beatriz Fernández, C. Derrick Quarles, Héctor González-Iglesias, Björn Meermann, Rosario Pereiro

**Affiliations:** † Department of Physical and Analytical Chemistry, University of Oviedo, Julián Clavería 8, 33006 Oviedo, Spain; ‡ Division 1.1 − Inorganic Trace Analysis, Federal Institute for Materials Research and Testing (BAM), Richard-Willstätter-Str. 11, 12489 Berlin, Germany; § Elemental Scientific, Inc., 7277 World Communications Drive, Omaha, Nebraska 68122, United States; ∥ Instituto de Productos Lácteos de Asturias, Consejo Superior de Investigaciones Científicas (IPLA-CSIC), 33300 Villaviciosa, Spain

## Abstract

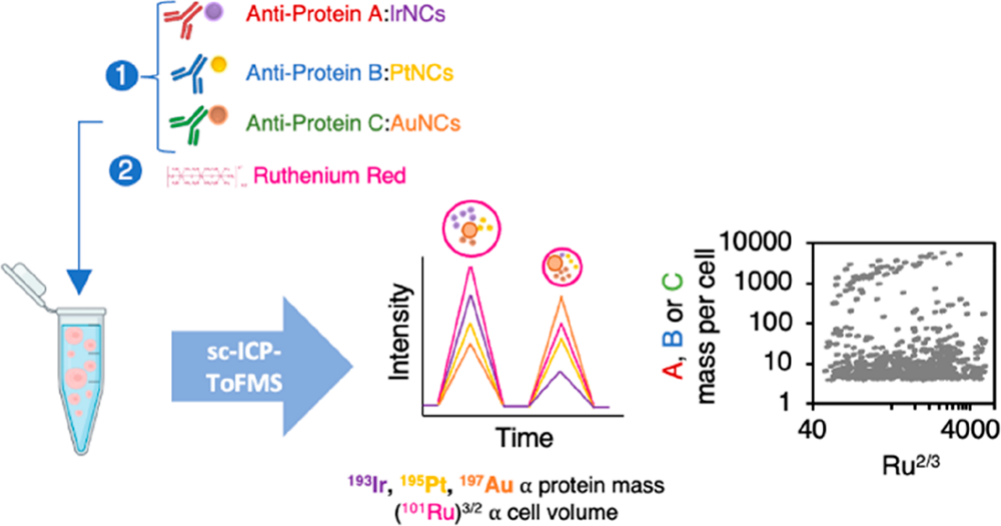

An automated and straightforward detection and data treatment strategy for the determination of the protein relative concentration in individual human cells by single cell–inductively coupled plasma–time-of-flight mass spectrometry (sc-ICP-ToF-MS) is proposed. Metal nanocluster (NC)-labeled specific antibodies for the target proteins were employed, and ruthenium red (RR) staining, which binds to the cells surface, was used to determine the number of cell events as well as to evaluate the relative volume of the cells. As a proof of concept, the expression of hepcidin, metallothionein-2, and ferroportin employing specific antibodies labeled with IrNCs, PtNCs, and AuNCs, respectively, was investigated by sc-ICP-ToF-MS in human ARPE-19 cells. Taking into account that ARPE-19 cells are spherical in suspension and RR binds to the surface of the cells, the Ru intensity was related to the cell volume (i.e., the cell volume is directly proportional to (Ru intensity)^3/2^), making it possible to determine not only the mass of the target proteins in each individual cell but also the relative concentration. The proposed approach is of particular interest in comparing cell cultures subjected to different supplementations. ARPE-19 cell cultures under two stress conditions were compared: a hyperglycemic model and an oxidative stress model. The comparison of the control with treated cells shows not only the mass of analyzed species but also the relative changes in the cell volume and concentration of target proteins, clearly allowing the identification of subpopulations under the respective treatment.

## Introduction

The heterogeneous nature of cells implies that cells of the same line may differ in the levels of their metal and biomolecule expression by up to 2 or 3 orders of magnitude.^1^ It has also been reported that such significant cell-to-cell variations may be the origin of several pathologies.^2^ Therefore, the correct interpretation for the expression of target analytes in cell populations can be difficult to assess unless samples are analyzed on a quantitative cell-to-cell basis. Furthermore, it is known that cellular transcriptome is also affected by the cell volume,^3,4^ thus it is convenient to evaluate individual cell volumes when analyzing target analytes in cell populations. This is particularly important in cellular models where the cells are subjected to different treatments; if not considered, the studied biological phenomena could be concealed or incorrect conclusions could be drawn. Therefore, in addition to the mass of protein per cell^5^ or the number of protein molecules per cell,^6,7^ it is of high interest to know the concentration of specific proteins in each cell.

Single cell–inductively coupled plasma–mass spectrometry (sc-ICP-MS) is a promising technique for the study of endogenous elements in cells as well as specific biomolecules by using metal-labeled antibodies.^8,9^ Additionally, a new generation of time-of-flight (ToF) mass analyzers has allowed for the simultaneous detection of several target analytes within single cells.^10−12^ While works related to the analysis of human cells using sc-ICP-ToF-MS are scarce, examples can be found in the literature where this technique has been applied to elemental fingerprinting in algae,^13^ investigating metal uptake by yeast,^14^ and conducting multielement analysis in sperm.^15^ For protein analysis, the selection of the antibody (Ab) labels must offer the maximum possible sensitivity, as proteins are generally on the order of fg or ag per cell. Typically, Maxpar polymers are employed to obtain metal-labeled antibodies, being the number of detectable atoms per Ab, or about 100–140 atoms.^16^ In this context, the use of metal nanoclusters (MNCs) offers a higher amplification, on the order of hundreds or thousands of metal atoms per Ab (e.g., 579 and 1760 metal atoms for AuNCs and IrNCs, respectively, have been reported).^17,18^

In order to determine the protein concentration in individual cells by sc-ICP-ToF-MS, the measurement of a proper volume marker for each cell is required. The selection of a proper ICP-MS detectable cell volume marker is not a straightforward step. Previous works have shown that some endogenous elements such as Mg and Ca could be related to the cells’ volume.^11,19,20^ However, it is quite challenging to simultaneously measure the very low concentrations of endogenous elements and the metal labels (significant *m*/*z* difference). An alternative strategy was proposed by Rapsomaniki et al.^21^ using a Ru complex which covalently binds to the amino groups of proteins, but this methodology is limited to certain cellular models where cells are exposed to some stressors. Ideally, the volume marker should label just the cell membrane so that it can be related to the cell volume regardless of the supplementation effect. In this vein, cell membranes can be labeled with ruthenium red (RR) as proposed by Qin et al.^22^ for the analysis of yeast strains and algal species. This strategy was successfully used to relate Ru signals and signals from intrinsic elements in single cells (Mg and P) to the cell volume. However, the procedure requires one to calculate the absolute volume, which is quite cumbersome, and microscopic measurements must be carried out for each cell model. Furthermore, it is not possible to measure exactly the same cells by ICP-ToF-MS and microscopy.

For a comparison of cell cultures subjected to different supplementations, the determination of the individual relative volume for each cell would provide crucial information. In this work, we present for the first time a straightforward strategy for the determination of the protein relative concentration of individual human cells by sc-ICP-ToF-MS. MNC-labeled specific antibodies for the target proteins were employed, and RR staining was used as a volume marker. An automated and simple detection approach to comparing cell populations was established by measuring labeled proteins and the ^101^Ru^+^ intensity signal by ICP-ToF-MS. As a case study, the expression of three target proteins was investigated by sc-ICP-ToF-MS in human ARPE-19 cells, a cell line of the retinal pigment epithelium (RPE), under two stress conditions: a hyperglycemic model culturing the cells with high glucose (GL) concentration and an oxidative stress model treating the cells with 2,2′-azobis(2-methylpropionamidine) dihydrochloride (AAPH). Cells were subjected to a multiplexed immunoassay using IrNCs, PtNCs, and AuNCs to label specific antibodies to hepcidin (HP), metallothionein-2 (MT2), and ferroportin (FPN), respectively, and then the same cells were stained with RR. Thus, in the present study the target proteins were simultaneously quantified on a cell-to-cell basis by sc-ICP-ToF-MS, providing a new perspective of cell heterogeneity to in vitro cellular studies.

## Experimental Section

Details related to the reagents employed, the conditions used for the culture and incubation of ARPE-19 cells, the supplementation treatments of ARPE-19 cells with AAPH or GL, and the synthesis of the MNC immunoprobes are collected in the Supporting Information (SI).

## Methods

### Immunoassay with ARPE-19 Cells and MNC-Labeled Immunoprobes

Fixated cell suspensions were subjected to an immunoassay to simultaneously label the three proteins of interest with the MNC-labeled immunoprobes. The immunoassay protocols used to label HP, MT2, and FPN in ARPE-19 cells were optimized, following the procedure proposed in previous works,^1,5^ in terms of immunoprobe concentration (referring to the Ab concentration) to ensure the total recognition of the proteins and the number of washing steps to avoid nonspecific interactions. The protocols were independently performed with the three immunoprobes (Anti-h-HP:IrNCs, Anti-h-MT2:PtNCs, or Anti-h-FPN:AuNCs), and optimized Ab concentrations were found as follows: 4 μg mL^–1^, 10 μg mL^–1^, and 4 μg mL^–1^, respectively. Figure S1 in the SI displays the experimental results obtained by sc-ICP-ToF-MS for the analysis of control (CT) ARPE-19 cells labeled with Anti-h-HP:IrNC, Anti-h-MT2:PtNC, or Anti-h-FPN:AuNC immunoprobes using different Ab concentrations. After the immunoassay, ARPE-19 cells were tagged with RR in suspension. For such a purpose, cells were incubated for 30 min with a 50 μg mL^–1^ RR solution. Afterward, the cellular pellet was washed twice with phosphate-buffered saline (PBS; 0.1 M at pH 7.4) to remove excess RR.

### sc-ICP-ToF-MS Analysis and Data Processing

Cells were introduced into the sc-ICP-ToF-MS instrument suspended in 50 mM Trizma buffer (pH 7.4) at a 1 × 10^5^ cells mL^–1^ concentration. The selection of the adequate concentration of ARPE-19 cells in suspension was performed using a serial dilution with CT cells in the range of 1 × 10^4^–1 × 10^6^ cells mL^–1^ (data not shown). A multielemental standard solution (containing Pt, Ir, Au, and Ru) was employed for ionic calibration, with six points ranging from 0 to 5 ng mL^–1^. Two suspensions were analyzed daily to determine the transport efficiency (TE) of the experimental setup for sc-ICP-ToF-MS: a commercial PtNPs standard and a solution of CT ARPE-19 cells. A citrate-stabilized PtNP standard (46 ± 3 nm, NanoComposix) was measured at a particle concentration of 1 × 10^5^ NP mL^–1^. The TE using the PtNP standard was found to be 81 ± 3% within the same day (*n* = 5). However, TE for sc-ICP-ToF-MS calculations was determined using cell suspensions (51 ± 4% within the same day; *n* = 5). Event discrimination was performed with TOFpilot software (Tofwerk), and Excel (Microsoft) and JASP programs (box plots and mass frequency histograms) were also employed for data treatment. ICP-ToF-MS was tuned with STDS mode to measure the different cellular labels (^101^Ru^+^, ^193^Ir^+^, ^195^Pt^+^, and ^197^Au^+^), whereas CCTS mode was employed for the detection of endogenous elements. Optimized operating conditions are collected in Table S1. For confirmation of the sc-ICP-ToF-MS methodology based on MNC-labeled immunoprobes and RR tagging, the average concentration of HP and FPN proteins in CT- and GL-treated ARPE-19 cells was also determined with commercial ELISA kits in a cytoplasmic fraction of cell lysates. ARPE-19 cells were centrifuged, resuspended in 10 mM Tris-HCl at pH 7.4, and lysed to separate the cytoplasmic and membranous fractions by ultrasonication. Supernatants obtained by centrifugation (15 700*g* for 30 min at 4 °C) were stored at −80 °C until they were used in the ELISA assays.

### Instrumentation

For sc-ICP-ToF-MS measurements, an ICP-ToF 2R (Tofwerk) coupled to a microFAST Single Cell System (Elemental Scientific, Inc.) for sample introduction was employed. Such a sample introduction system includes an autosampler, a CytoNeb 50 nebulizer (90 psi at 50 μL min^–1^), a CytoSpray chamber, and a one-piece ICP-MS torch. Cell counting in ARPE-19 suspensions (fixated cells and resuspended in Trizma) was done with a BD Accuri C6 cytometer (BD Biosciences). Optical images of the cells suspensions were acquired using an optical microscope (Leica DM IL LED). Ultrasonication (Bandelin sonoplus HD2070 probe) was performed for protein determination with ELISA kits.

## Results and Discussion

### Tagging of ARPE-19 Cells with Ruthenium Red: Cell Discrimination and Volume Marker

For the analysis of biomolecules by sc-ICP-MS using metal-labeled antibodies, it would be very convenient to monitor both the elemental label and a cell intrinsic element (e.g., Ca, Cu, Fe, P, etc.) to confirm the integrity of the cells as well as the proper Ab recognition.^1,5−7^ However, the mass difference between the common endogenous cell elements (low *m*/*z* range) typically at very low levels and the metals employed for the labels (e.g., noble metals or lanthanides in a high *m*/*z* range) can be a limitation to simultaneously detecting all of them with high sensitivity by sc-ICP-ToF-MS. In our experiments, the simultaneous measurement of an intrinsic element together with the sensitive detection of Ir, Pt, and Au from the MNC-labeled immunoprobes was attempted with the ToF-MS analyzer to obtain a fingerprint of each individual cell. Nevertheless, if the instrument was tuned to favor the low masses (CCTS mode for the detection of Fe), then there was not enough sensitivity to detect the MNC labels and vice versa. Thus, RR, which is a salt that binds to the polysaccharides of the cell membrane,^22^ was employed for detecting individual ARPE-19 cells. The mass difference between Ru and the MNC labels makes it possible to tune the ICP-ToF-MS to simultaneously detect Ru, Ir, Pt, and Au in the ARPE-19 cell suspension with the proper sensitivity.

After the cells were labeled with MNC immunoprobes, they were tagged with RR and measured by sc-ICP-ToF-MS. The compound Poisson threshold (α = 0.001) was used to discriminate cell events. As depicted in Figure S2, the ARPE-19 cell suspension showed two different types of events for the time-resolved profile: type 1 events, where either Ru, Ir, Pt, or Au appeared individually, and type 2 events, where Ru was simultaneously detected with combinations of one, two, or three MNC labels. The intensity of Ru was significantly larger (*p* value = 1 × 10^–40^, *t* test 95% confidence level) in type 2 events than in type 1 events. This fact suggests that events where only Ru was detected probably correspond to membrane fragments from ARPE-19 cells that broke during the preparation process or the nebulization. This was also the case for the intensities observed for Ir, Pt, and Au in type 1 events: signals detected for MNC labels were always significantly smaller than those observed in type 2 events (*t* test 95% confidence, *p* value = 0.004, 2 × 10^–22^, and 0.002 for Ir, Pt, and Au, respectively). This fact can be attributed to the presence of free MNC-labeled immunoprobes not bound to the proteins. In previous works using quadrupole mass analyzers, such free immunoprobes were successfully discriminated by applying a Poisson threshold or a 5σ threshold.^1,5^ However, such strategies do not fit the ToF data,^23^ and an alternative strategy was proposed to discriminate cell events: only events where Ru was simultaneously detected with at least one of the labels from MNC-labeled immunoprobes were considered.

Following such premises, the cellular TE value employed for calculations was determined using ARPE-19 cell suspensions. TE was calculated as the ratio of detected cell events by sc-ICP-ToF-MS (i.e., detection of Ru simultaneously with at least one Ab label) over the number of introduced cells (the same cell suspensions were previously measured by flow cytometry). Cellular TE was found to be 51 ± 4% (five replicates). Here, it must be highlighted that this value may be underestimated: it is possible that ARPE-19 cells with low concentrations for the target proteins, where only Ru was detected, have not been considered as a cell event with the proposed criterion.

**Figure 1:**
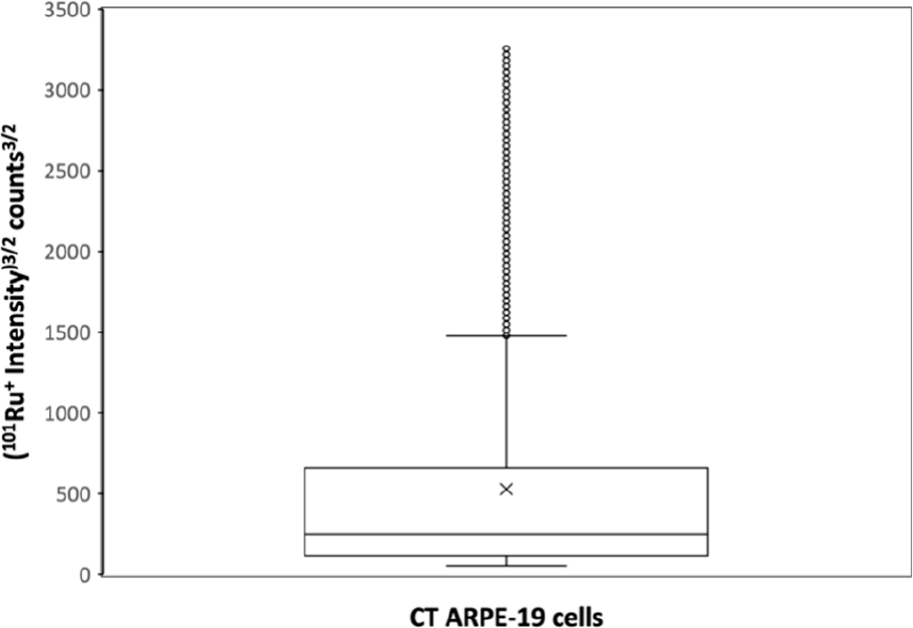
Box plot representing the (^101^Ru^+^ intensity)^3/2^ value measured in the cellular event by sc-ICP-ToF-MS for a suspension of CT ARPE-19 cells. The graph was constructed with the ^101^Ru^+^ intensity signals from CT ARPE cells of the GL treatment (data from four biological replicates with three instrumental replicates each). The boxes represent the interquartile region, the lower and upper whiskers are *Q*_0_ and *Q*_4_, respectively, the lines within the boxes indicate the median, and the crosses indicate the average value.

In this work, the RR was used not only to determine the number of cell events but also to evaluate the relative volume of the cells. A new strategy is proposed here using the Ru intensity signal to get relative concentrations of the target proteins within each cell. Thus, taking into account that ARPE-19 cells are spherical in suspension and RR binds to the surface of the cells, the Ru intensity was related to the cell volume, cell volume ∝ (Ru intensity)^3/2^,^22^ making it possible to determine not only the mass of the target proteins in each individual cell but also the relative concentration. The mass of protein per cell (*M*_p_c__) was calculated with eq 1, where *I*_e_ is the intensity of the elemental label (MNCs), *F* is the flow rate, η_c_ is the cellular TE, *t* is the integration time, MW_p_ is the molecular weight of the protein, *b*_e_ is the slope of the elemental calibration curve, AWe is the atomic weight of the label, and *A* is the amplification provided by each immunoprobe.^5^ Furthermore, the relative concentration of the protein in each cell (*C*_p_c__) was directly obtained with the ratio between the mass of the protein and the cell volume following eq 2, where Ru_i_ is the intensity of ^101^Ru^+^ in each individual cell. This simple approach allows the comparison of concentrations of target proteins between cells from different batches (e.g., CT and AAPH or GL-treated).
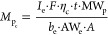
1
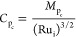
2Additionally, the RR was employed to identify multiple cell events. On one hand, ARPE-19 cells were studied in suspension using an optical microscope (representative images are shown in Figure S3). Cells were randomly selected from 30 images to measure their diameter, which was found to be 16 ± 4 μm (*n* = 500). The smallest cell diameter was 10 μm, and the largest was 34 μm. This means that the volume of the largest cell was about 40 times larger than the volume of the smallest cell. On the other hand, when ARPE-19 cell suspensions were measured by sc-ICP-ToF-MS, the Ru intensity signal was directly obtained from the time-resolved profiles and could be used to calculate the cells volume. As an example, Figure 1 depicts the box plot constructed for the (^101^Ru^+^ intensity)^3/2^ value obtained for CT ARPE-19 cells measured by sc-ICP-ToF-MS. The minimum value obtained for cells was 55 counts^3/2^, whereas the maximum value was 3250 counts^3/2^ (median 250 counts^3/2^). Note that 96% of the cells in the suspension have a volume variation in the range observed by microscopy measurements (only 4% of the cells exhibited a larger volume), and thus cell events whose (^101^Ru^+^ intensity)^3/2^ value was more than 40 times the minimum value were considered to be multiple events and were discarded for data evaluation.

### Study of Protein Levels in Stressed ARPE-19 Cells by sc-ICP-ToF-MS

As a case of study, the expression of HP, MT2, and FPN was investigated by sc-ICP-ToF-MS in cultured ARPE-19 cells under two different conditions: a hyperglycemic model stressing the cells with GL (100 mM for 48 h) and an oxidative stress model treating the cells with AAPH (5 mM for 1 h). The sc-ICP-ToF-MS analyses allowed for the cell-by-cell simultaneous detection of HP, MT2, and FPN and the determination of their relative concentration. For such purpose, after discriminating the cell events from the background by applying the selected threshold, the ^193^Ir^+^, ^195^Pt^+^, and ^197^Au^+^ intensity signals were transformed into absolute masses of Ir, Pt, and Au by the external calibration (Methods section). The mass of metals for each cell was then converted into the corresponding protein mass following a previously reported protocol.^5^ Finally, relative concentrations (expressed as the protein mass per relative cell volume) in each cell were obtained using the (^101^Ru^+^ intensity)^3/2^ value measured for each cell.

According to studies reported by Gundlach-Graham et al.,^23^ limits of detection (LoDs) for ToF-MS with fast analog-to-digital conversion (ADC)-based detection must be calculated by considering a compound Poisson distribution of the background signal. Thus, ^193^Ir^+^, ^195^Pt^+^, and ^197^Au^+^ background signals were measured from time-resolved profiles of CT ARPE-19 cells according to such criteria. LoDs were calculated using the equation proposed by Gundlach–Graham for ADC signals, and then the intensities were transformed into the mass of proteins with eq 1. LoDs were found to be 3.8 ± 0.4 ag/cell for HP, 9 ± 1 ag/cell for MT2, and 4.4 ± 0.6 fg/cell for FPN.

### Effect of Hyperglycemia on ARPE-19 Cells Treated with Glucose

sc-ICP-ToF-MS was used to evaluate the possible changes in the HP, MT2, and FPN levels in individual ARPE-19 cells after hyperglycemia induced by high GL concentration. Differences observed between CT- and GL-treated cells were studied by applying the *t* test, and the results are collected in Table 1 (including the*t* test for ^101^Ru^+^ intensity, the mass of protein per cell, and the concentration of protein in terms of the mass per cell volume). As can be observed, GL treatment affected the average mass of each protein per cell when considering the whole ARPE-19 cell population (*n* = 14 171 and 15 461 cellular events for CT- and GL-treated cells, respectively), observing an overexpression for the three proteins with GL treatment. However, when comparing relative protein concentrations, no significant differences in the mean value were obtained.

**Table 1:** *t-*Test Results Obtained for the Analysis of ARPE-19 Cells after the Immunoassay with MNC-Labeled Immunoprobes and RR Tagging by sc-ICP-ToF-MS, with a Comparison of CT Cells and Treated Cells^a^^,^^b^

	**Glucose Treatment**			**AAPH Treatment**		
	**df**	***p* Value**	**Observation GLvsCT**	**df**	***p* Value**	**Observation AAPH** vs **CT**
^**101**^**Ru**^**+**^**Intensity (cts)**	29 232	0.04	>	6369	2 × 10^–53^	>
**Mass of HP (ag/cell)**	12 182	0.02	>	4381	0.04	>
**Concentration of HP (ag/cell volume)**	12 182	0.13	=	4381	0.005	<
**Mass of MT2 (ag/cell)**	19 356	1 × 10^–15^	>	6484	0.3	=
**Concentration of MT2 (ag/cell volume)**	19 356	0.08	=	6484	1 × 10^–70^	<
**Mass of FPN (fg/cell)**	2655	0.002	>	1021	0.02	>
**Concentration of FPN (fg/cell volume)**	2655	0.05	=	1021	0.05	=

a Hyperglycemia (GL treatment) and oxidative stress (AAPH treatment).

b Differences in the average values between CT and treated-cells populations were determined applying a *t* test at 95% confidence for variables with unequal variances. Data included the analysis of four biological replicates in all cases, each of them with three instrumental replicates. Degrees of freedom, df; cell volume = (^101^Ru^+^ intensity)^3/2^ value; >, overexpression in treated cells; <, underexpression in treated cells, and =, no difference between CT and treated cells.

**Figure 2:**
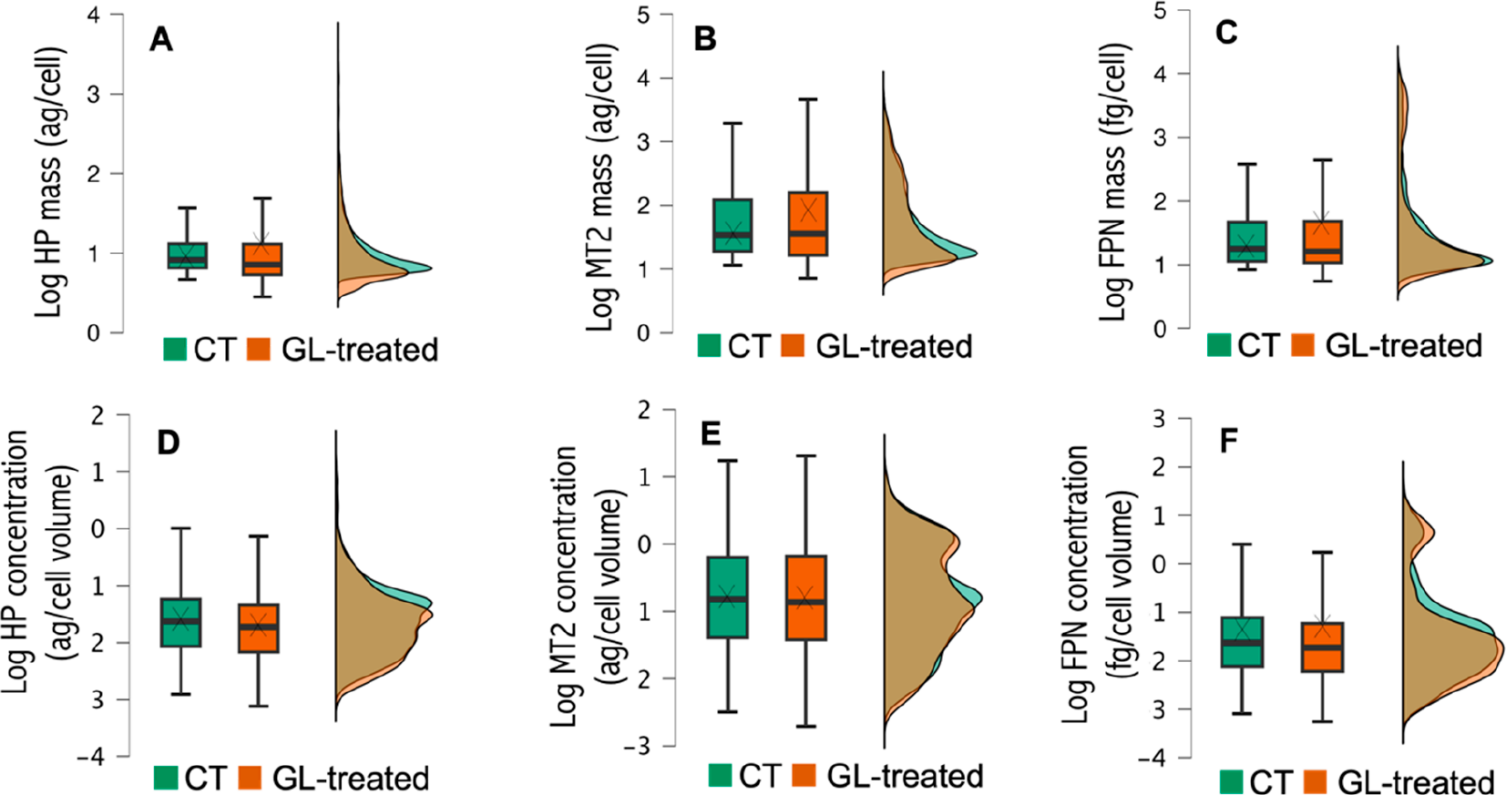
Box plots and mass frequency histograms (in percentage) representing the mass of protein per cell (panels A–C) and the relative protein concentration (panels D–F) obtained by sc-ICP-ToF-MS for HP, MT2, and FPNN in CT- (in green) and GL-treated ARPE-19 cells (in orange). (A, D) Hepcidin. (B, E) Metallothionein-2. (C, D) Ferroportin. Data included the analysis of four biological replicates for CT- and GL-treated ARPE-19 cells, each of them with three instrumental replicates.

Panels A–C of Figure 2 outline box plots comparing the distribution of the mass of HP, MT2A, and FPN per cell for CT- and GL-treated populations. The following observations can be noticed: mean values (x symbol in the graphs) were significantly larger for the GL-treated cells, whereas the median values were not affected by the treatment. However, the mass of the proteins per cell in GL-treated cells followed a more dispersed distribution both above and below the median (note that Figure 2 uses a logarithmic scale). Therefore, comparing just population averages (as done with conventional methods such as commercial ELISA kits) can mislead regarding the effect of the treatment. For example, if only the mean mass of the specific proteins is taken into account, then it seems that the GL treatment increases the levels of the three target proteins. Nevertheless, evaluating the suspensions on a cell-by-cell basis, it was observed that the GL treatment broadens the distribution on both extremes, indicating a higher variability of the masses of HP, MT2, and FPN within cell populations. Regarding the relative concentrations of HP, MT2, and FPN (Figure 2D–F, respectively), the differences in broadness observed between CT- and GL-treated cells decrease. Additionally, the box plots related to the distribution of the mass of the proteins per cell (panels A–C) were skewed: the dispersion above the median was larger than that below it. However, such skewness was no longer noticed when the cell volume was taken into account (panels D–F), suggesting that the dispersed values corresponded to cells that have larger amounts of protein but also a larger volume. The histograms obtained for HP, MT2, and FPN are also collected in Figure 2, representing the frequency of cells (expressed as a percentage) which contain a certain protein mass (panels A–C) or protein relative concentration (panels D–F). There was a single wide population for both CT- and GL-treated cells when the volume was not considered, whereas two different populations appeared (two maxima can be seen in the histograms, especially for MT2 and FPN) when accounting for cell volume.

**Figure 3:**
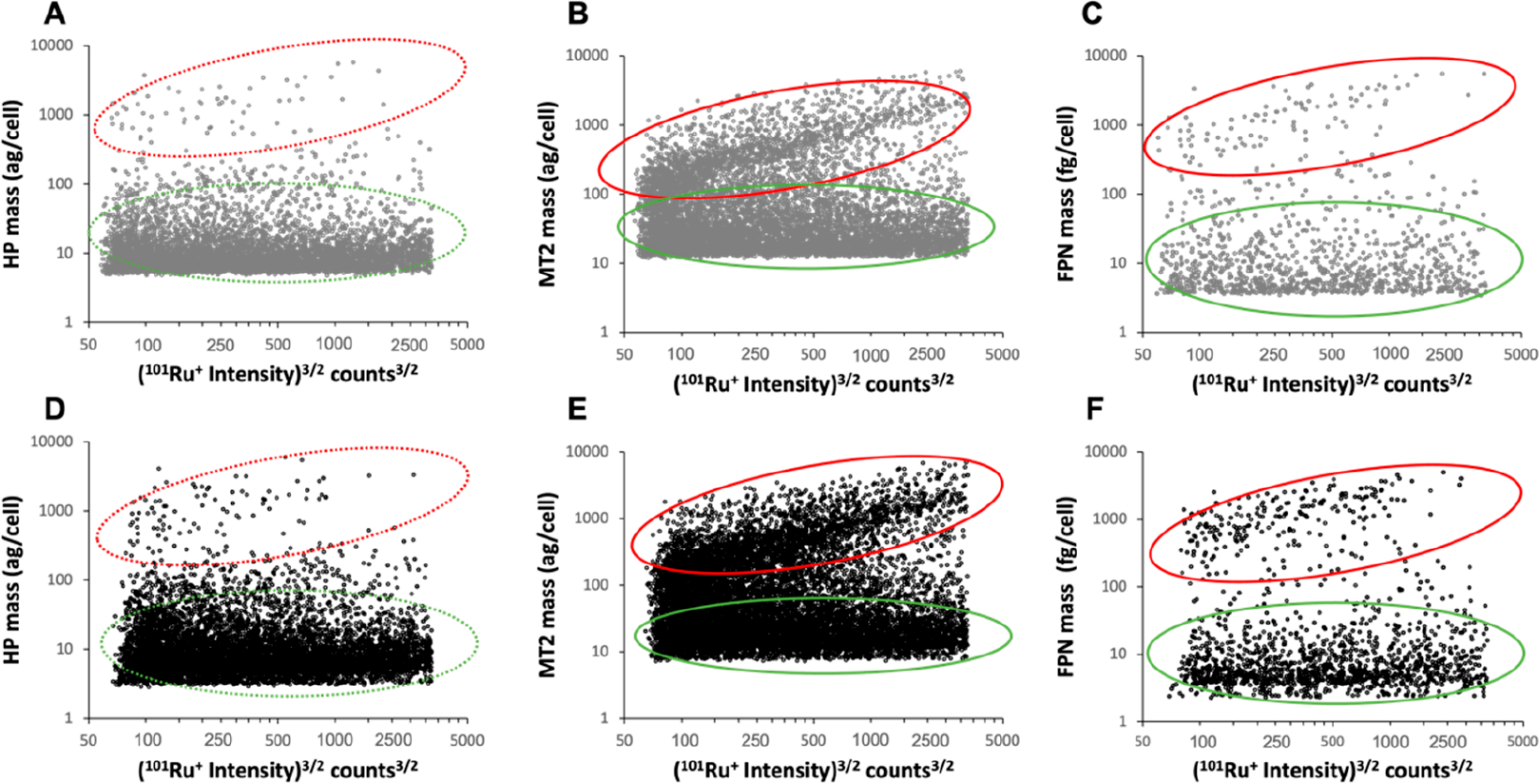
Scatter plots representing the protein mass per cell versus the cell volume obtained by sc-ICP-ToF-MS for HP, MT2, and FPNN in CT- and GL-treated ARPE-19 cells. Panels A–C correspond to CT cells, whereas panels D–F collect the scatter plots for GL-treated cells. ^101^Ru^+^ signals were simultaneously measured in the cells with metals from MNC-labeled immunoprobes. Red ellipses mark the cellular population with a larger protein mass, whereas the green ellipses mark the group with a lower protein mass.

The correlation between the mass of the proteins and the cell volume was also studied by constructing scatter plots (Figure 3). The two cellular groups observed in the histograms of Figure 2 (panels D–F) can also be identified in the scatter plots: cellular populations with a larger protein mass were highlighted in red in the upper part of the graphs, whereas the cells with a lower protein mass were marked in green at the bottom. In the case of MT2 and FPN in CT ARPE-19 cells (Figure 3; panels B, C), there was a linear increasing correlation between the volume of the cells and the protein mass for the population denoted with the red circle: the larger the cell volume, the higher the mass of the protein. Two populations were also observed for HP (Figure 3; panel A), although the protein mass linear correlation with volume was not noticeable for this protein. When treating the cells with GL (Figure 3; panels D–F), the same trends were observed, but a higher percentage in the red marked group was noticed for MT2 and FPN. Therefore, it could be stated that after the GL treatment there is a higher number of cells whose volume is linearly related to the mass of MT2 and FPN.

To evaluate whether the cell volume was also affected by the treatment, ^101^Ru^+^ intensity signals were also studied for the whole population in CT- and GL-treated cells. Figure S4 shows the frequency histogram obtained by sc-ICP-ToF-MS representing the percentage of cells that have a certain cell volume (i.e., (^101^Ru^+^ intensity)^3/2^ value). For larger cells, the same distribution was observed for CT- and GL-treated cells. However, for smaller cells, a different trend was clearly found: for up to 65 cts^3/2^, only CT cells were observed (i.e., GL-treated cells were not found at this interval), whereas a higher number of CT- compared to GL-treated cells was found in the range of 65–140 cts^3/2^. As can be also observed in Table 1, the average ^101^Ru^+^ intensities were significantly larger for the GL-treated cells (0.04 *p* value), indicating that hyperglycemia increases the cells volume. A high GL concentration may affect the cell size by increasing the average protein content and therefore the cell volume, as occurred in yeast and mammalian cells.^24^ In addition, hyperglycemia induces oxidative stress, lipid peroxidation, and apoptosis and inhibits cell proliferation,^25,26^ which may alter the levels of antioxidants and proteins controlling metal homeostasis such as MT2, HP, and FPN.^27^

To confirm the validity of the proposed strategy, experimental results obtained for HP and FPN (mass of the protein/cell) through the analysis of ARPE-19 cells by sc-ICP-ToF-MS were compared with those measured in lysates from CT- and GL-treated cells employing commercial ELISA kits. Rather than comparing the absolute protein mass, it is more appropriate to study the tendencies found between the two groups (CT- and GL-treated) employing both methodologies because different cell populations (independently subcultured depending on the experiments) as well as different antibodies (which may have different specificities) were employed for ELISA and sc-ICP-ToF-MS analyses. Three biological samples (six instrumental replicates each) were analyzed with the ELISA kits, and the average mass of HP and FPN per cell was overexpressed in GL-treated cells, with an *n*-fold change of 1.4 in both cases. Additionally, significant differences were found between CT- and GL-treated cells by applying a *t* test at 95% confidence (5 × 10^–4^*p* value for HP and 2 × 10^–6^*p* value for FPN). The same tendencies were found by sc-ICP-ToF-MS where *n*-fold changes between GL-treated and CT cells were, respectively, found to be 1.4 and 1.3 for HP and FPN and significant differences were found between CT- and GL-treated populations (Table 1). Therefore, the results obtained agree with the technique commonly employed in cellular biology. However, it should be highlighted that with ELISA analyses only the average protein mass in the cell culture can be obtained, whereas the mass of the protein can be determined in each detected cell by sc-ICP-ToF-MS (not the mean value for the whole cell population), with it also being possible to account for the cell volume that allows us to better understand biological mechanisms underlying the cell stress response (e.g., increasing of the cells’ metabolism or increasing of the cells’ volume).

### Effect of Induced Oxidative Stress on ARPE-19 Cells Treated with AAPH

**Figure 4:**
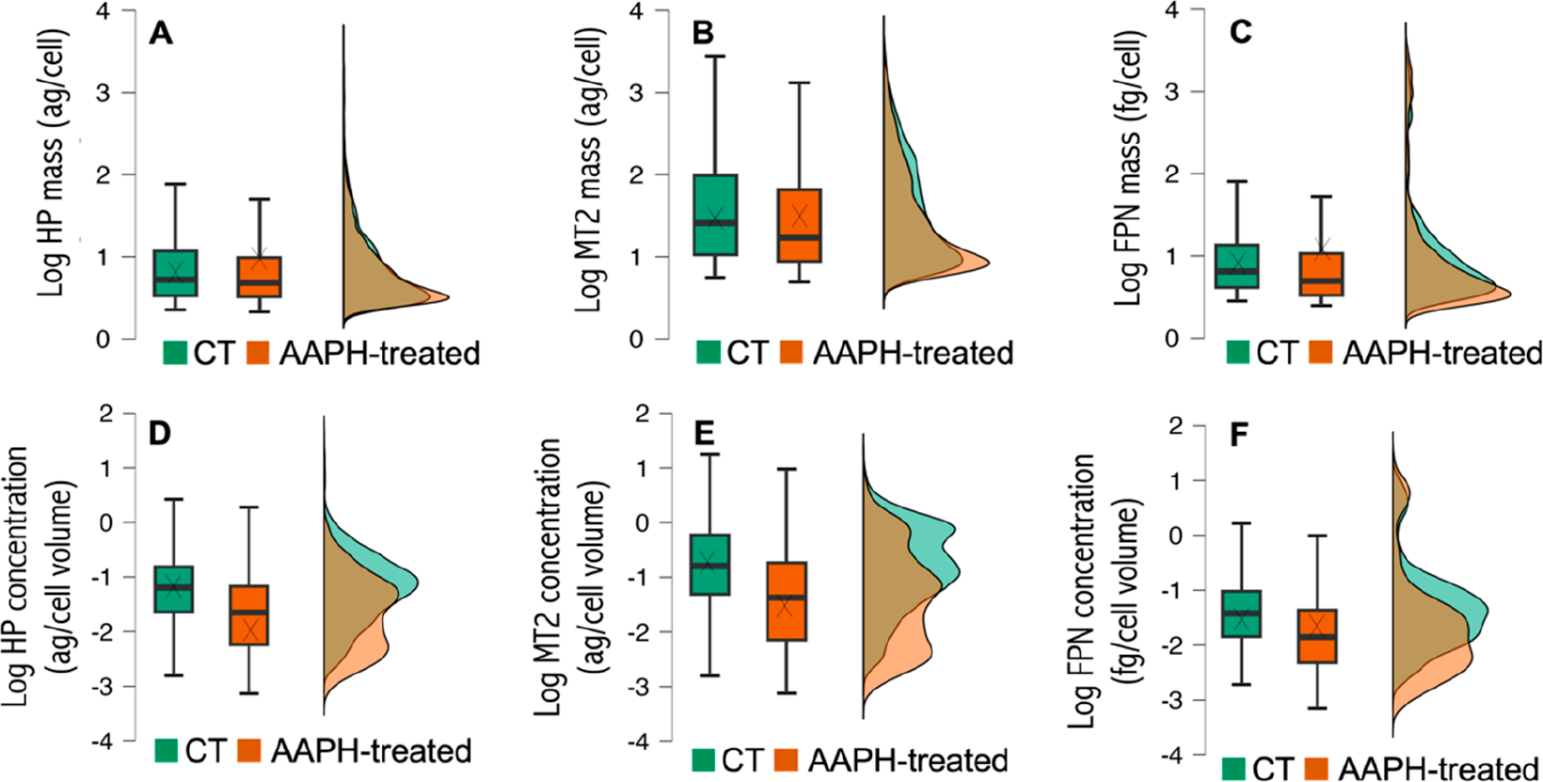
Box plots and mass frequency histograms (in percentage) representing the mass of protein per cell (panels A–C) and the relative protein concentration (panels D–F) obtained by sc-ICP-ToF-MS for HP, MT2, and FPN in CT (in green) and AAPH-treated ARPE-19 cells (in orange). (A, D) Hepcidin. (B, E) Metallothionein-2. (C, D) Ferroportin. Data included the analysis of four biological replicates for CT- and GL-treated ARPE-19 cells, each of them with three instrumental replicates.

The same method was employed to study the amounts of HP, MT2, and FPN in CT and AAPH-treated ARPE-19 cells. Table 1 shows the results obtained by applying the *t* test, and Figure 4 depicts the box plots and histograms comparing the distribution of the protein mass (panels A–C) and the proteins’ relative concentrations (panels D–F) for CT- and AAPH-treated populations. As can be seen in Figure 4 (panels A–C), the average mass of protein per cell was increased for HP and FPN when treating the cells with AAPH, while no significant differences were found for MT2 (the crosses for HP and FPN in the AAPH-treated cells are positioned at higher protein masses, and these differences are statistically significant as indicated in Table 1). Concerning the median of the protein mass per cell, it was not altered after AAPH treatment in the case of HP but decreased for MT2 and FPN (from 1.41 ag/cell to 1.23 ag/cell and from 0.81 fg/cell to 0.69 fg/cell, respectively). Taking into account the individual cell volume, the average relative protein concentrations were found to be downregulated for the three proteins in the box plots (Figure 4; panels D–F). However, the differences in FPN concentration between CT- and AAPH-treated ARPE-19 cells were not statistically significant (Table 1).

**Figure 5:**
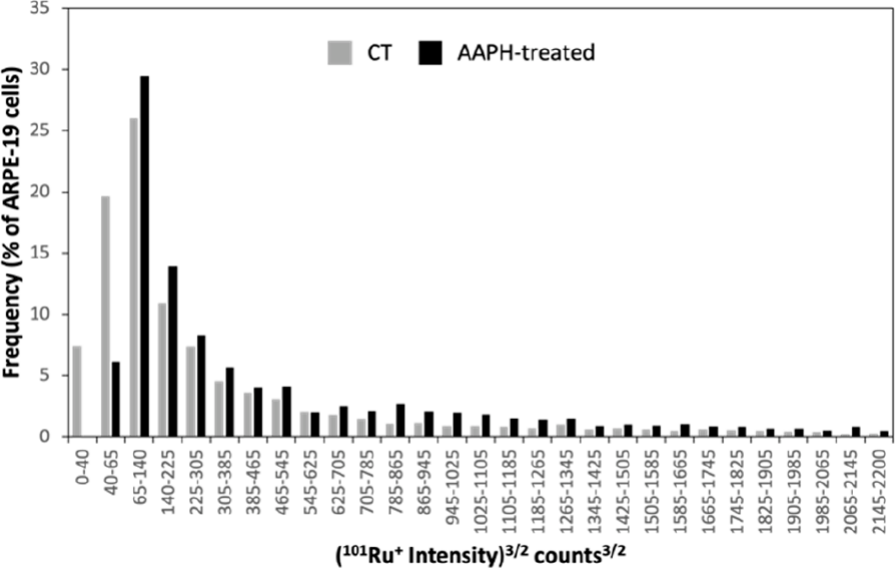
Cell volume frequency histogram (in percentage) for CT (in gray) and AAPH-treated ARPE-19 cells (in black) obtained by sc-ICP-ToF-MS after the immunoassay with MNC-labeled immunoprobes and RR tagging (*n* = 4635 and 3505 for CT- and AAPH-treated cells, respectively).

The mass frequency histograms depicted in Figure 4 (panels A–C) exhibited a single broad cell population for the three proteins for both CT- and AAPH-treated cells. However, when considering the cells’ volume (panels D–F), several size populations can be identified, and a different behavior was observed for CT- and AAPH-treated cells. The relative protein concentration histogram for HP in CT cells (Figure 4D) has one maximum, in contrast to AAPH-treated cells where two different cell groups could be identified. Regarding MT2 (Figure 4E), a higher percentage of cells was identified at low protein concentrations for AAPH-treated cells compared to that for CT (three cell groups were clearly identified after treatment). Two cell groups were always found for FPN (Figure 5F), but different percentages of cells at low and medium protein concentrations were found for CT- and AAPH-treated cells. The different cell populations observed by comparing CT- and AAPH-treated cells can also be identified by correlating the mass of the proteins and the cells’ volume. Figure S5 depicts the scatter plots for HP, MT2, and FPN. (Note that the whole cell population in this case was lower: 8140 cell events for AAPH treatment compared to 29 632 cell events for GL treatment.) Similar to that observed for GL treatment, two cellular groups were observed for low and high protein mass together with a linear increasing correlation between the volume of the cells and the high protein masses (especially for MT2 and FPN), though this effect is less noticeable.

Finally, Figure 5 depicts the frequency histogram obtained by sc-ICP-ToF-MS for CT- and AAPH-treated cells representing the percentage of cells that have a certain cell volume. Experimental results showed that a higher percentage of cells with high Ru signals (above 65 cts^3/2^) was found for AAPH-treated cells than for CT-treated cells, meaning a higher cell volume for the supplemented cells. It is well known that AAPH is a peroxyl radical generator increasing both the production of reactive oxygen species in the RPE^28^ and the relative cell volume by altering membrane permeability^29^ and increasing the intracellular water content. Therefore, the proposed strategy allows us to obtain interesting findings for AAPH treatment that can be achieved only by studying the cell population on a cell-by-cell basis and considering the individual cell volume. For example, a higher mass of HP and FPN per cell was observed in AAPH-treated cells than in CT-treated cells, but it was found that the treatment also increases the cell volume considerably; therefore, the higher HP and FPN masses correspond not only to higher protein concentrations inside the cell after the treatment but also to a larger cell size.

## Conclusions

The evaluation of the effect of cell culture supplementation requires knowing, on a cell-to-cell basis, the changes produced in the mass and the concentrations of the target species in each cell, as well as in the cell volume. The strategy presented here with sc-ICP-ToF-MS detection is based on the use of MNC-labeled antibodies as specific tags for protein determination and RR as a volume marker, thus allowing sensitive individual protein mass determination in single cells as well as providing the relative volume of each single cell and the relative target protein concentration inside the cell. The proposed automated and straightforward detection and data treatment approach enables the analysis of large data sets with reliable results and allows for an effective comparison between CT and treated cell cultures, shining new light on the consequences brought about by the treatment. It also offers the potential to evaluate the total mass of protein per single cell (provided that a proper metallic label is employed), granting a deeper understanding of the biochemical processes occurring within each cell.

## Supporting Information Available

sc-ICP-ToF-MS operating conditions, time-resolved profiles, optical image of the cells, and box plots and histograms of the protein distribution (PDF)
