# Supplementary material for: Single Cell–ICP–ToF-MS for the Multiplexed Determination of Proteins: Evaluation of the Cellular Stress Response
Source: Anal Chem. 2023 Aug 11;95(35):13322–9. doi: 10.1021/acs.analchem.3c02558 (PMC10483461; doi:10.1021/acs.analchem.3c02558)
Supplement: Supplementary file 1 — ac3c02558_si_001.pdf [file ac3c02558_si_001.pdf]

Supporting Information

Single cell-ICP-ToF-MS for Multiplexed Determination of Proteins – Evaluation of Cellular Stress Response

Paula Menero-Valdés[†], Michail I. Chronakis[‡], Beatriz Fernández[†*], C. Derrick Quarles Jr.[§], Héctor González-Iglesias[¥], Björn Meermann[‡], Rosario Pereiro[†]

[†]Department of Physical and Analytical Chemistry, University of Oviedo, Julián Clavería 8, 33006, Oviedo, Spain.

[‡]Division 1.1 – Inorganic Trace Analysis, Federal Institute for Materials Research and Testing (BAM), Richard-Willstätter-Str. 11, 12489 Berlin, Germany.

[§]Elemental Scientific, Inc. - 7277 World Communications Drive, Omaha, NE 68122, USA.

[¥] Instituto de Productos Lácteos de Asturias, Consejo Superior de Investigaciones Científicas (IPLA-CSIC), 33300, Villaviciosa, Spain.

*Corresponding authors email addresses: fernandezbeatriz@uniovi.es

Abstract

This Supporting Information contains some related to the *Experimental Section*, including reagents and experimental methods, as well as a Figure and Table with the optimized operating conditions for sc-ICP-ToF-MS analyses. Concerning the *Results and Discussion Section*, different Figures are included showing experimental results related to the optimization of the antibody concentration in the MNCs–immunoprobes, a time resolved profile for the analysis of CT ARPE-19 cells, optical images of ARPE-19 cells in suspension, and the analysis of HP, MT2 and FPN by sc-ICP-ToF-MS.

Table of Content

1. EXPERIMENTAL SECTION **2**
1.1. Chemicals and Materials **2**
1.2. Experimental Methods **3**
1.2.1. Cell Line and Culture Conditions. 3
1.2.2. Synthesis and Labelling of Specific Antibodies with Metal Nanoclusters. 3
1.2.3. Immunoassay with ARPE-19 Cells and MNCs–labelled Immunoprobes 3
1.2.4. sc-ICP-ToF-MS Analysis and Data Processing. 6
2. RESULTS AND DISCUSSION **7**
2.1. Tagging of ARPE-19 Cells with Ruthenium Red: Cells Discrimination and Volume Marker **7**
2.2. Study of Protein Levels in Stressed ARPE-19 Cells by sc-ICP-ToF-MS **8**
3. REFERENCES **10**

1. EXPERIMENTAL SECTION

1.1. Chemicals and Materials

$IrCl_3$·x H_2O (99.9% crystals, Sigma-Aldrich), $AuCl_4Na$·x H_2O (99% powder; Sigma-Aldrich) and H_2PtCl_6 (8%*wt*, Sigma-Aldrich) were employed as metal precursors for the synthesis of IrNCs, AuNCs and PtNCs, respectively. Lipoic acid (98% powder, Across Organics) and tri-sodium citrate dihydrate (99% crystalline powder, Merck) were used as surface ligands and $NaBH_4$ (98% powder, Sigma-Aldrich) was selected as the reducing agent. MNCs were bioconjugated to Anti-h-Hepcidin (Anti-h-HP; ab30760, Abcam), Anti-h-Ferroportin (Anti-h-FPN; LS-C179226, LSBio) and Anti-h-Metallothionein-2 (Anti-h-MT2; PAB868Hu01, Cloud Cone Corp) using 1-ethyl-3-(3-dimethylaminopropyl) carbodiimide (98% powder, Across Organics) and N-hydroxysuccinimide (>98% powder, Sigma-Aldrich). Amicon ultracentrifugal filter units (Merck) with 3 and 100 kDa pore size were employed for MNCs and MNCs–labelled immunoprobes (Ab:MNCs) purification, respectively.

ARPE-19 cells (ATCC) were cultured in Corning® cell culture flasks. Cells grew in Dulbecco's modified eagle medium/nutrient mixture F-12 (DMEMF12, Sigma-Aldrich) supplemented with 10% (*v/v*) inactivated fetal bovine serum (Gibco, Thermo-Fischer Scientific) and 1% (*v/v*) penicillin/streptomycin (P/S; Gibco, Thermo-Fischer Scientific) and in hybridoma serum free medium (Gibco, Thermo-Fischer Scientific) with 5% (*v/v*) L-glutamine (Gibco, Thermo-Fischer Scientific) and 1% (*v/v*) P/S supplementation. ARPE-19 cells were respectively subjected to oxidative stress and hyperglycemia stress conditions with AAPH (Sigma-Aldrich) and D-(+)-glucose (99.5%, Sigma-Aldrich), both diluted in supplemented hybridoma. Cells were detached from the flasks using trypsin EDTA (Gibco, Thermo-Fischer Scientific) and fixated with paraformaldehyde (PFA; VWR Chemicals).

The immunoassays with ARPE-19 cells in suspension using MNCs–labelled immunoprobes were performed employing phosphate buffered saline (0.1 M at pH 7.4) (PBS) as washing solution, triton X-100 (Sigma-Aldrich) to permeabilize cellular membranes and goat serum (Sigma-Aldrich) and bovine serum albumin (BSA; 99% powder, Merck) to block non-specific interactions. Cell membrane was tagged with ruthenium red (Sigma Aldrich). Trizma hydrochloride (99.9% crystals, Sigma-Aldrich) was used as buffer for sc-ICP-ToF-MS analyses.

Transport efficiency for the sample introduction system was daily checked with a citrate stabilized PtNPs standard (46 ± 3 nm, NanoComposix). Ru, Ir, Au and Pt calibrations were performed with ICP

standards (1000 μg mL^{-1}, Sigma-Aldrich) in 3% ultrapure HNO_3 (LabKem). Deionized ultrapure water, resistivity 18.2 MΩ·cm (Purelab Flex 3&4; ELGA-Veolia, High Wycombe) was employed throughout. The average concentration of two proteins in ARPE-19 cells was also determined by commercial ELISA kits from MyBioSource for HP (MBS164980) and FPN (MBS164980).

1.2. Experimental Methods

1.2.1. ***Cell Line and Culture Conditions.*** ARPE-19 cells were cultured in T75 Corning® flasks in an incubator (37 °C and 5% CO_2) with supplemented DMEMF12 and, once confluent, media was changed to hybridoma. After 24 h, cells were non-treated (control cells, CT) or treated with AAPH (5 mM for 1 h) or GL (100 mM for 48 h) in hybridoma medium. AAPH and GL concentrations and culture time were selected based on previous studies with RPE cells *in vitro*.[1,2] Four biological replicates per condition were used: CT cells (both for GL and AAPH treatments), GL–treated cells and AAPH–treated cells. Cells analyzed by sc-ICP-ToF-MS were then subjected to a mild fixation with PFA and stored in PBS until further use.

1.2.2. ***Synthesis and Labelling of Specific Antibodies with Metal Nanoclusters.*** IrNCs, PtNCs and AuNCs were synthesized and labelled with specific antibodies using previously optimized protocols.[3-] Taking into account the amplification provided by each immunoprobe (i.e., the average number of metal atoms per Ab: 1760 for Ir, 1194 for Pt and 579 for Au) as well as the proteins concentration expected in RPE cells, Anti-h-HP was labelled with IrNCs, Anti-h-MT2 with PtNCs and Anti-h-FPN with AuNCs. Ab:MNCs molar ratios employed for the labelling processes were 1:10, 1:20 and 1:5 for IrNCs, PtNCs and AuNCs, respectively. Thus, three MNCs–labelled immunoprobes were synthesized for the detection of target proteins: Anti-h-HP:IrNCs, Anti-h-MT2:PtNCs and Anti-h-FPN:AuNCs.

1.2.3. ***Immunoassay with ARPE-19 Cells and MNCs–labelled Immunoprobes.*** The immunoassays with MNCs–labelled immunoprobes were performed employing PBS as washing solution, Triton X-100 to permeabilize cellular membranes, and goat serum and bovine serum albumin to block non–specific interactions. After the immunoassay protocol, cells suspensions were diluted to $1 \cdot 10^5$ cells mL^{-1} in 50 mM Trizma® hydrochloride adjusting pH to 7.4 with hydrochloric acid. It is worth noting that our group previously conducted a study where different solvents (Milli–Q, NaCl, and Trizma) were investigated for ensuring RPE cells integrity when introducing them into the ICP. The experimental results revealed that the cellular transport efficiency with human RPE cells was significantly higher when using the Trizma buffer.[6] The immunoassay protocols to label the three

target proteins in ARPE-19 cells were optimized in terms of immunoprobe concentration (referred to the antibody concentration) to ensure the total recognition of the proteins and the number of washing steps to avoid non–specific interactions. The protocols were independently performed with the three immunoprobes (Anti-h-HP:IrNCs, Anti-h-MT2:PtNCs or Anti-h-FPN:AuNCs). **Figure S1** displays the experimental results obtained by sc-ICP-ToF-MS for the analysis of HP, MT2A and FPN in CT ARPE-19 cells labelled with Anti-h-HP:IrNCs (1-5 μg mL^{-1} Ab concentration), Anti-h-MT2:PtNCs (2-10 μg mL^{-1} Ab concentration) or Anti-h-FPN:AuNCs (1-5 μg mL^{-1} Ab concentration) immunoprobes.

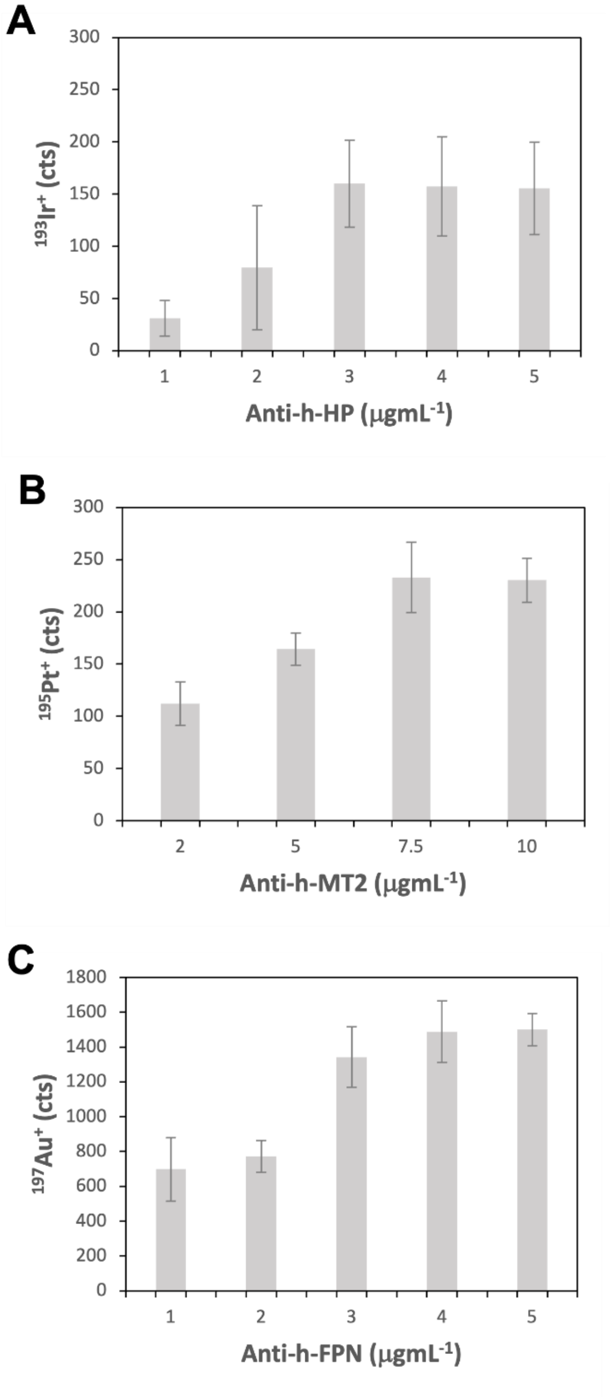

Figure S1. Immunoassay optimization for the determination of HP, FPN and MT2 in CT ARPE-19 cells by sc-ICP-ToF-MS using Anti-h-HP:IrNCs, Anti-h-MT2:PtNCs and Anti-h-FPN:AuNCs immunoprobes. The protocol was independently performed with the three immunoprobes and different Ab concentrations were evaluated in each case. A) Hepcidin, B) Metallothionein-2, and C) Ferroportin. The graphs depict the concentration of the metal labels per cell. Uncertainties represent the standard deviation from the mean of three replicates.

1.2.4. sc-ICP-ToF-MS Analysis and Data Processing.

Table S1 collects the optimized operating conditions for the analysis of endogenous elements and MNCs–labels in ARPE-19 cells by sc-ICP-ToF-MS.

Table S1. sc-ICP-ToF-MS operating conditions. The instrument was tuned with STDS mode to measure Ru, Ir, Pt and Au. CCTS mode was employed for endogenous element detection.

Parameter	Value
Sample flow rate (μL min^{-1})	10
Plasma power (w)	1550
Gas flow rate (L min^{-1}):	
○ Plasma gas	14
○ Additional gas	0.65
○ Nebulizer gas	0.27
Integration time (ms)	3
Acquisition time (s)	180
CCT Mass (V)	• STDS mode: 270
CCT Bias (V)	• STDS mode: -2
H_2 flow rate – CCT (mL min^{-1})	• STDS mode: 0
Analysis time per sample (min)	• 10

2. RESULTS AND DISCUSSION

2.1. Tagging of ARPE-19 Cells with Ruthenium Red: Cells Discrimination and Volume Marker

Figure S2 depicts the time resolved profile obtained for ARPE-19 cells after the immunoassay with MNCs–labelled immunoprobes and RR tagging by sc-ICP-ToF-MS. Two different types of events have been observed, denoted with numbers 1 and 2 in the Figure. Type 1 corresponds to cellular event where Ru, Ir, Pt or Au appeared individually, whereas Type 2 is associated to events where Ru was simultaneously detected with combinations of one, two or the three MNCs labels.

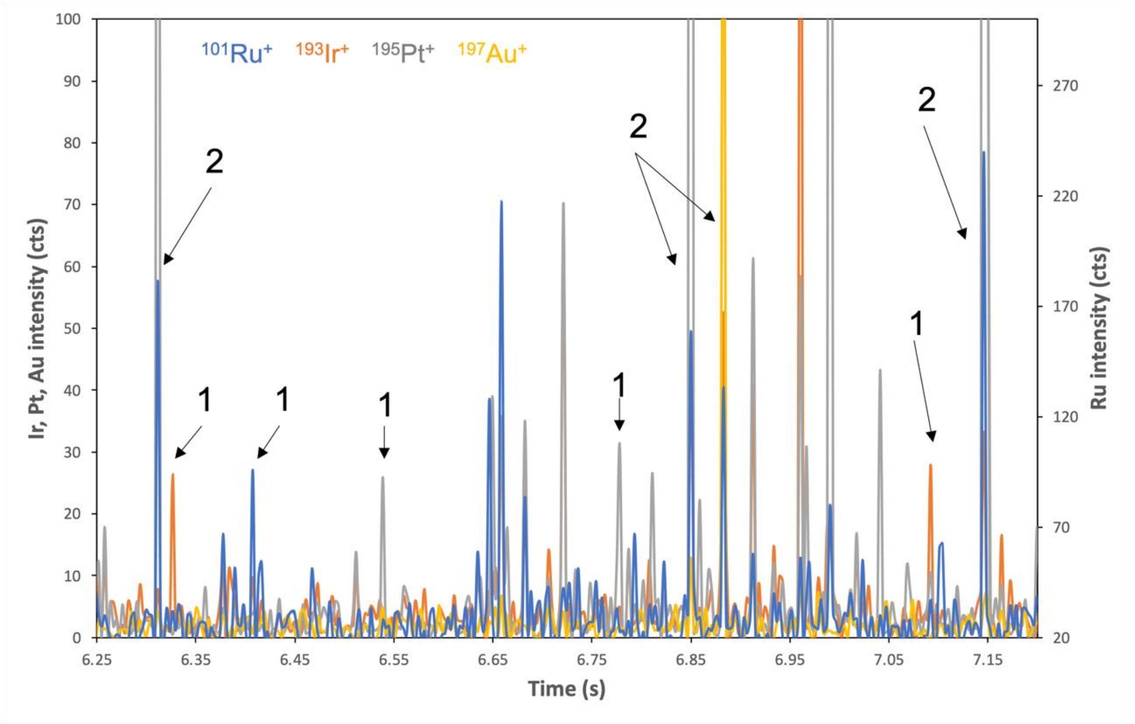

Figure S2. Time resolved profile obtained for ARPE-19 cells after the immunoassay with MNCs–labelled immunoprobes and RR tagging by sc-ICP-ToF-MS. Two different type of events in the same cell suspension can be observed denoted as Type 1 (number 1 in the profile: Ru, Ir, Pt or Au appeared individually) and Type 2 (number 2 in the profile: Ru was simultaneously detected with combinations of one, two or the three MNCs labels).

ARPE-19 cells were measured by microscopy (in suspension) to determine their dimensions. Cells were randomly selected from 30 images to measure their diameter, which was found to be 16 ± 4 µm (n=500). As an example, **Figure S3** shows two optical images obtained for ARPE-19 cells.

A) B)

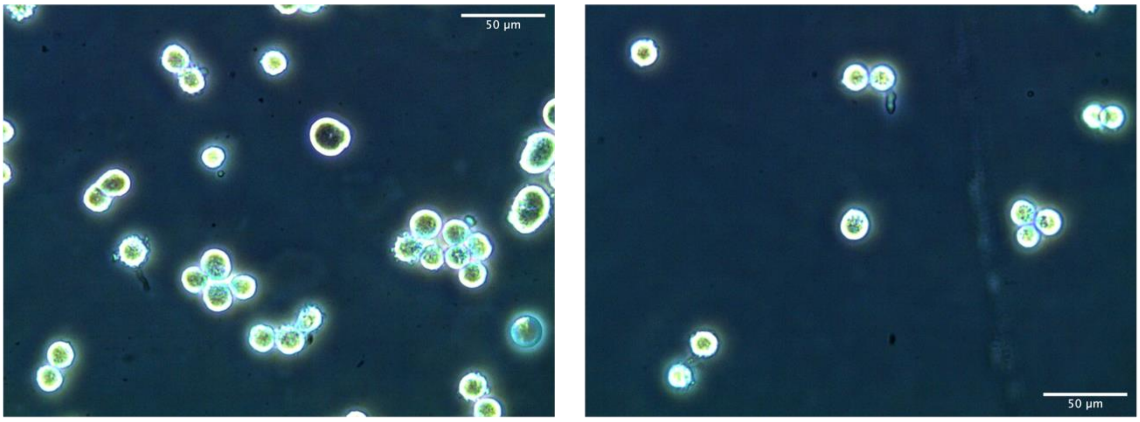

Figure S3. Phase contrast images obtained by using an optical microscope for ARPE-19 cells. Suspension of CT ARPE-19 cells (without being submitted to the immunoassay with MNCs–labelled immunoprobes and RR tagging ($1 \cdot 10^5$ cells mL^{-1} in PBS).

2.2. Study of Protein Levels in Stressed ARPE-19 Cells by sc-ICP-ToF-MS

In order to evaluate the cell volume in CT and GL–treated ARPE-19 cells, $^{101}Ru^+$ intensity signals were measured by sc-ICP-ToF-MS for the whole population in the two groups of cells. **Figure S4** shows the frequency histogram obtained representing the percentage of cells that has a certain cell volume, i.e., $(^{101}Ru^+ \text{ intensity})^{3/2}$ value.

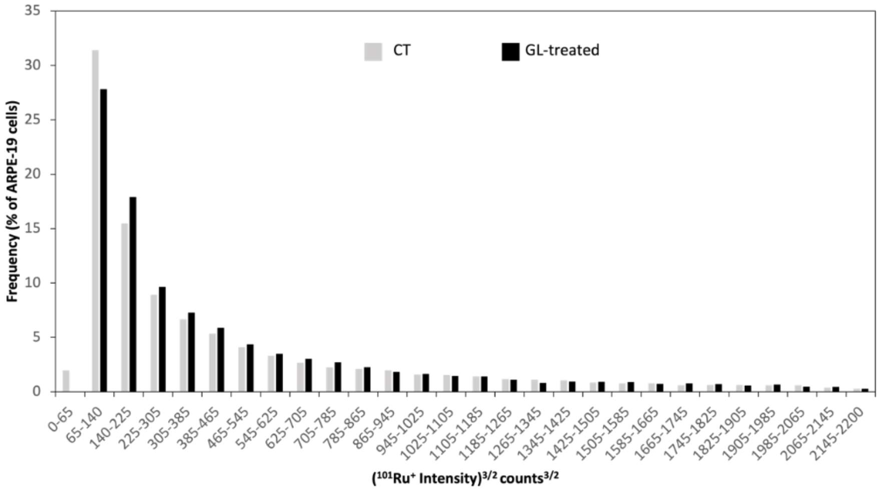

Figure S4. Cell volume frequency histogram (in percentage) for CT (in grey) and GL–treated ARPE-19 cells (in black) obtained by sc-ICP-ToF-MS after the immunoassay with MNCs–labelled immunoprobes and RR tagging (n=14171 and 15461 for CT and GL–treated cells, respectively).

Finally, the correlation between the mass of the proteins and the cells volume was studied in CT and AAPH–treated cells by constructing scatter plots. **Figure S5** depicts the scatter plots for HP, MT2 and FPN: A-C panels for CT cells and D-F panels for AAPH–treated cells. Similar to that observed for GL treatment, two cellular groups were observed for low and high protein mass together with a linear increasing correlation between the volume of the cells and the high protein masses (especially for MT2 and FPN), though this effect is less noticeable.

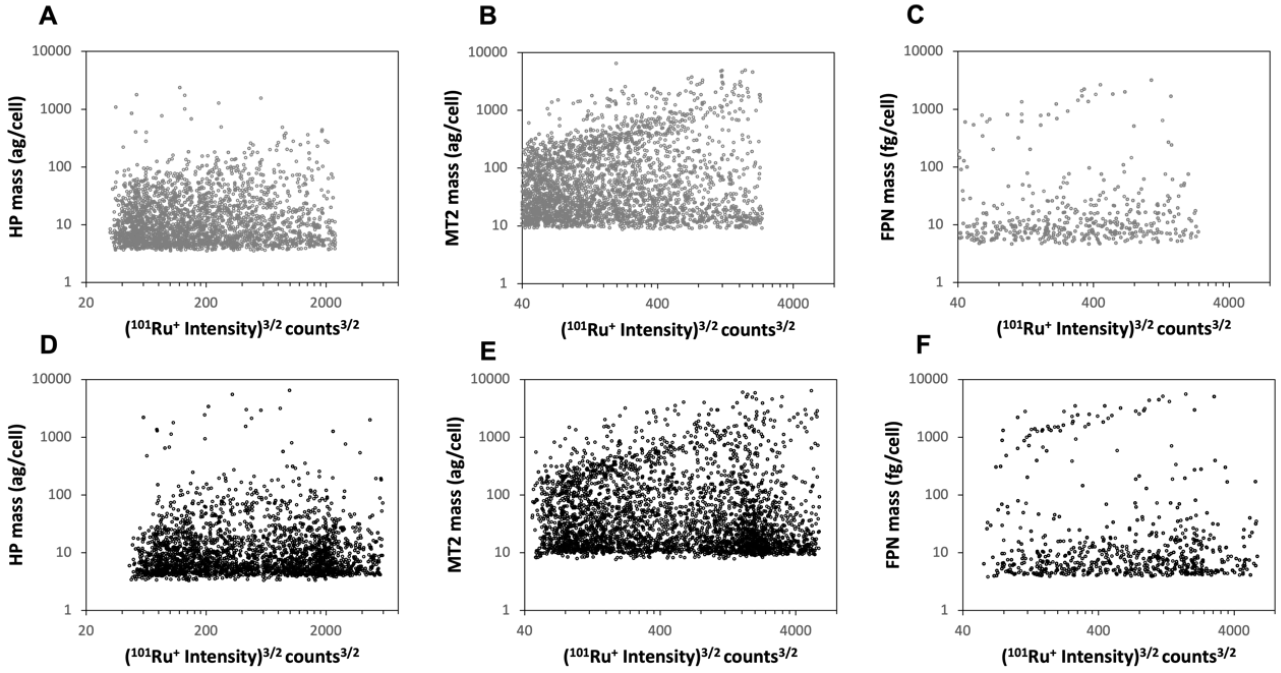

Figure S5. Scatter plots representing the protein mass per cell versus the cell volume obtained by sc-ICP-ToF-MS for HP, MT2 and FPN in CT and AAPH–treated ARPE-19 cells. A-C panels correspond to CT cells, whereas D-F panels collect the scatter plots for AAPH–treated cells.

3. REFERENCES

[1] Rodríguez-Menéndez, S.; García M.; Fernández, B.; Álvarez, L.; Fernández-Vega-Cueto, A.; Coca-Prados, M.; Pereiro, R.; González-Iglesias, H. *Nutrients* **2018**, 10,1874.

[2] González de Vega, R.; García, M.; Fernández-Sánchez, M.L.; González-Iglesias, H.; Sanz-Medel, A. *Metallomics*, **2018**, 10, 83–92.

[3] Cruz-Alonso, M.; Fernández, B.; García M.; González-Iglesias, H.; Pereiro, R. *Analytical Chemistry* **2018**, 90, 12145–12151.

[4] Menero-Valdés, P.; Lores-Padín, A.; Fernández, B.; González-Iglesias, H.; Pereiro, R. *Talanta* **2022**, 244, 123424.

[5] Lores-Padín, A.; Cruz-Alonso, M.; González-Iglesias, H.; Fernández, B.; Pereiro, R. *Microchimica Acta* **2019**, 186, 705.

[6] Lores-Padín, A.; Mavrakis, E.; Fernández, B.; García, M.; González-Iglesias, H.; Pereiro, R.; Pergantis, S.A. *Analytica Chimica Acta*, **2022**, 1203, 339701.
